# Predicting Outcomes of Atezolizumab and Bevacizumab Treatment in Patients with Hepatocellular Carcinoma

**DOI:** 10.3390/ijms241411799

**Published:** 2023-07-22

**Authors:** Ji Won Han, Jeong Won Jang

**Affiliations:** 1 The Catholic University Liver Research Center, College of Medicine, The Catholic University of Korea, Seoul 06591, Republic of Korea; tmznjf@catholic.ac.kr; 2 Division of Gastroenterology and Hepatology, Department of Internal Medicine, College of Medicine, Seoul St. Mary’s Hospital, The Catholic University of Korea, Seoul 06591, Republic of Korea

**Keywords:** hepatocellular carcinoma, atezolizmumab-bevacizumab, biomarker

## Abstract

A combination of atezolizumab with bevacizumab (AB) is the first regimen that has shown superiority compared to sorafenib and is now being used as the systemic treatment of choice for hepatocellular carcinoma (HCC) patients with Barcelona Liver Cancer Clinic stage C. However, a considerable number of patients do not achieve survival or significant responses, indicating the need to identify predictive biomarkers for initial and on-treatment decisions in HCC patients receiving AB. In this manuscript, we summarized the current data from both experimental and clinical studies. This review will be beneficial for both clinicians and researchers in clinical practice as well as those designing experimental, translational, or clinical studies.

## 1. Introduction

Hepatocellular carcinoma (HCC) ranks as the sixth most common cancer and the third leading cause of cancer-related deaths worldwide [1]. The prevalence of this cancer is expected to increase by 55% from 2020 to 2040 [2]. Even though surgical and locoregional treatments can be used in some cases, it is estimated that systemic therapies might be the chosen treatment for 50–60% of HCC patients [1]. Multi-targeted tyrosine kinase inhibitors (TKIs) including sorafenib, lenvatinib, regorafenib, and cabozantinib, which target various molecules, can be used as first- or later-line systemic treatments. These agents commonly target the vascular endothelial growth factor receptor (VEGFR) and also have various molecular targets depending on each drug. However, other than sorafenib and lenvatinib, no drugs have been approved for first-line systemic treatment of advanced HCC, as trials have not demonstrated a significant clinical benefit compared to sorafenib.

Recent breakthroughs have led to a new era in systemic therapies, as immune-checkpoint inhibitors (ICIs) have proven to be effective in patients with HCC. However, the use of ICIs as a monotherapy has demonstrated limited efficacy with a response rate between 15% and 20%, which benefits only a small subgroup of HCC patients in a second-line setting. There are several suggested mechanisms related to the resistance to ICI treatment in HCC, including tumor-intrinsic and extrinsic factors [3]. Thus, numerous efforts to overcome this resistance and improve the clinical outcome of HCC patients have been made, and combinations of other regimens to ICIs have also been tried.

In 2020, the results of the IMbrave150 trial, which enrolled 501 treatment-naïve patients with advanced HCC and assigned them randomly to receive either atezolizumab combined with bevacizumab (AB) or sorafenib monotherapy, were published [4]. AB is the first agent that has shown superiority compared to sorafenib as a first-line systemic treatment, and can be administered at a dose of atezolizumab 1200 mg plus bevacizumab 15 mg/kg IV every 3 weeks, with target concentrations of 6 μg/mL for atezolizumab [5] and 140 μg/mL for bevacizumab [6]. The AB group exhibited a median progression-free survival (PFS) of 6.8 months, whereas the sorafenib group had a median PFS of 4.3 months. At the 12-month follow-up, 67.2% of patients in the AB group and 54.6% in the sorafenib group survived. The objective response rate (ORR) was 33.2% in the AB group, and 13.3% in the sorafenib group, which represented an improved, but insufficient clinical benefit.

Atezolizumab is a monoclonal antibody of the IgG1 isotype that acts on PD-L1 [7], which is present in immune cells or tumor cells within the tumor, blocking its interaction with receptors on the programmed cell death protein 1 (PD-1) and B7-1 (CD80) [4]. The interactions between PD-L1 and PD-1 inhibit T cell proliferation, cytokine secretion, and cytotoxic action, leading to T cell de-activation or exhaustion [8]. Atezolizumab reactivates tumor-specific cytotoxic T cells by disrupting the interaction between PD-1 and PD-L1. Bevacizumab is a monoclonal antibody of IgG1 isotype that targets VEGF, which is a key factor in angiogenesis [7]. Angiogenesis is the process of new blood vessel formation and is regulated by a balance between pro- and anti-angiogenic factors. Representative pro-angiogenic factors encompass the VEGF family, angiopoietins, epidermal growth factors (EGFs), and fibroblast growth factors (FGFs) [9]. Inflammatory cytokines such as interleukin (IL)-6 and IL-8 also participate in angiogenesis [10]. Anti-angiogenic therapies facilitate vascular normalization, impede tumor blood supply, and induce hypoxia and nutrient deficiency, consequently resulting in tumor cell death. Furthermore, they enable a more efficient delivery of therapeutic agents and immune cells to the tumor site. However, there is no randomized trial showing the clinical benefits of bevacizumab monotherapy in HCC.

The AB combination treatment may possess a potential synergistic effect in cancer treatment, enhancing their combined therapeutic efficacies. Anti-VEGF therapies counteract VEGF-induced immunosuppression within tumors and their microenvironments, potentially boosting anti-PD-1 and anti-PD-L1 effectiveness by reversing VEGF-driven immunosuppression and enhancing T cell infiltration, thereby enhancing antitumor immune responses [11].

**Figure 1:**
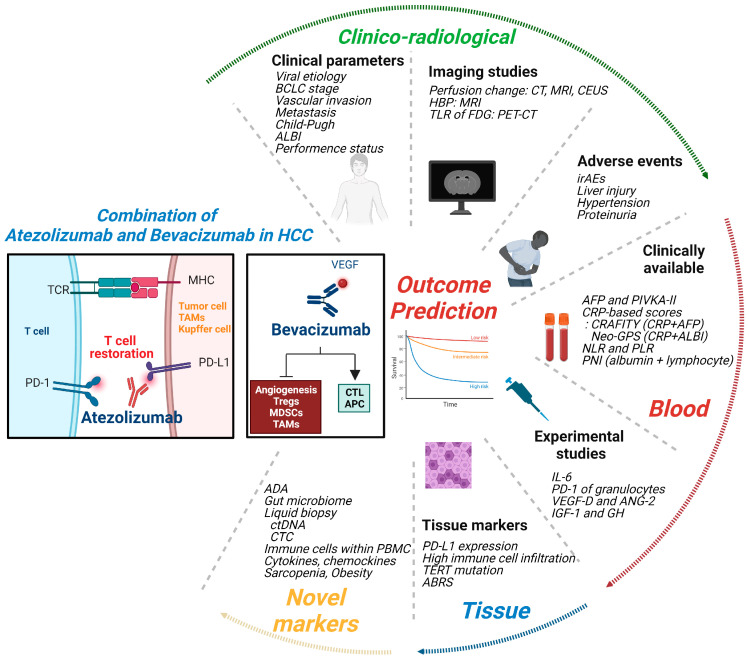
Predicting clinical outcomes in patients with HCC receiving Atezolizumab and Bevacizumab combination treatment. Atezolizumab targets PD-L1 resulting in the inhibition of PD-1 mediated T cell exhaustion pathway and T cell restoration. Bevacizumab targets VEGF and inhibits aberrant angiogenesis of tumor, as well as improving immunosuppressive TME via affecting Tregs, MDSCs, and TAMs. Predicting clinical outcomes of AB treatment can be performed by analyzing clinical factors such as medical records, laboratory tests, imaging tests, and the presence of AEs. They are also able to be performed by blood examinations, which include tumor markers, inflammatory markers, cytokines, and angiogenic factors. Liquid biopsy is also under investigation. Tissue studies include PD-L1 staining, whole genome or RNA sequencing, and staining for immune cell infiltration. Gut microbiome, CTCs, analysis for PBMCs, and various cytokines and chemokines should also be studied.

Although the combination regimen targets two different molecules, programmed cell death-ligand 1 (PD-L1) and vascular endothelial growth factor (VEGF), previous reviews have only focused on biomarkers for ICIs, and have not considered the point that this regimen further targets VEGF signaling. In this manuscript, we aim to reconcile the current knowledge on this topic and review both experimental/translational and clinical studies. This review will be beneficial for both clinicians and researchers in terms of making decisions and planning clinical, translational, and experimental studies. Brief introductions of the mechanisms of action and currently reported biomarkers for AB treatment in HCC patients are presented in Figure 1.

## 2. Clinico-Radiological Parameters

### 2.1. Clinical Parameters

#### 2.1.1. Etiology

Previous studies that have identified clinical factors associated with outcomes following AB treatment are summarized in Table 1. A recent experimental study showed that pathologic CD8+PD-1+ T cells might be associated with the limited role of anti-PD-1 treatment in NASH-related HCC [12]. Another study showed that hepatitis B virus (HBV)-infected subjects have distinct upregulation of peripheral blood inflammatory cytokine profiles, compared to the other etiologies including hepatitis C virus (HCV), NASH, and alcoholic liver diseases, suggesting different peripheral, intrahepatic, and intratumoral immune environments across the etiologies of HCC [13]. The tendency of better clinical outcomes in patients with viral etiologies have been reported in association with nivolumab [14], cabozantinib plus atezolizumab [15], and tremelimumab plus durvalumab [16] regimens.

In updated efficacy and safety data from IMbrave150, AB treatment in patients with viral etiologies including HBV and HCV had superior OS and PFS compared to those treated with sorafenib, although the subgroup analysis that only included AB treatment was not presented [17]. A recent meta-analysis, which included 3 large randomized phase III trials of nivolumab, AB, and pembrolizumab, suggested that the ICI regimen might be superior to sorafenib in terms of OS in HBV- and HCV-related HCC [12]. In addition, a recent network meta-analysis showed that patients with viral etiology showed significant survival benefits with the AB regimen compared to the TKIs [18]. However, such research has provided glimpses into the role of etiology as a predictive marker because these studies only showed the benefits of the AB regimen compared to the TKIs.

A reduction in AFP levels (≥75%) at 6 weeks following the start of therapy can serve as a potential biomarker for HCC patients receiving the AB treatment to predict improved OS and PFS, particularly in those with HBV etiology, but not in the HCV and non-viral etiologies in the recent report analyzing 440 patients who were included in the Phase Ib and III trials of IMbrave150 [19]. A small-sized (n = 66) recent real-world study also showed that patients with viral etiologies have better OS and PFS than patients with non-viral etiology [20]. Another small-sized retrospective study (n = 23) also showed that patients with viral etiology have higher ORR than those with non-viral etiology receiving the AB treatment [21]. However, other real-world studies did not find differences between the two groups, therefore larger nationwide studies are needed to validate the previous data. In addition, whether there might be a difference between HBV and HCV in the clinical outcome of AB treatment and its related mechanism also needs to be clarified.

#### 2.1.2. Tumor Burden

A larger tumor burden is associated with the more immunosuppressive tumor microenvironment (TME) contributed by regulatory T cells (Tregs), tumor-associated macrophages (TAMs), and myeloid-derived suppressor cells (MDSCs), as well as immunosuppressive cytokines such as tumor growth factor-beta (TGF-β) and IL-10 [22]. In HCC patients treated with nivolumab, an intrahepatic tumor size of more than 10 cm was associated with poor OS and PFS [23]. However, there is a possibility that the VEGF inhibitor may have a complementary effect, and therefore, the impact of tumor burden on the clinical outcome of the AB treatment remains unclear. A recent retrospective report (n = 121) showed that macrovascular invasion was associated with poor OS in AB treatment [24]. In addition, the presence of extrahepatic spread was associated with poor PFS in AB treatment in a recent real-world study (n = 433) [25]. The accumulation of data from further studies will confirm this association.

#### 2.1.3. Liver Function Parameters

Although most clinical trials, including IMbrave150, enrolled patients with good liver function of Child–Pugh A, liver function status in real-world practice might be variable and can change dynamically from the baseline status. Liver function plays a critical role in determining the prognosis and treatment options for HCC patients, particularly in patients receiving the AB treatment. A small-sized retrospective study (n = 100) showed that patients with Child–Pugh B had comparable ORR compared to those with Child–Pugh A, but had shorter OS and PFS [26]. A recent real-world study (n = 66) also showed that Child–Pugh A was a significant favorable factor for OS [20]. In addition, there was no difference between Child A and B patients in ORR, but PFS and OS were significantly better in the Child A group in a retrospective study (n = 457) [27], which indicates that liver function might be related to the prognosis rather than reflecting the therapeutic efficacy of the AB treatment.

In addition to the Child–Pugh score, albumin–bilirubin (ALBI) grade is also an important indicator of liver function, and several studies have investigated the associations between ALBI and clinical outcomes in AB treatment. Recent small-sized retrospective studies have demonstrated that ALBI grade [28,29] and Eastern Cooperative Oncology Group [28] scores before treatment were independent factors in predicting OS or PFS. Another real-world study (n = 28) also showed that a lower baseline modified ALBI (mALBI) predicts better ORR, and a Child–Pugh score of five and mALBI grades 1 and 2a were significantly associated with the continuation of treatment [30]. In addition to its baseline status, a worsening ALBI score within 3 weeks after the AB treatment was also significantly associated with OS in a retrospective analysis (n = 69) [31]. It can be combined with another biomarker. A recent multicenter retrospective study (n = 426) showed that the combination of mALBI grade and AFP (mALF score) significantly predicted OS and PFS [32].

### 2.2. Pre-Treatment Radiologic Examinations

#### 2.2.1. Hepatobiliary Phase of Magnetic Resonance Imaging (MRI)

In gadoxetic acid-enhanced MRI, it has been suggested that the hepatobiliary phase could be an imaging biomarker for the identification of β-catenin mutations in HCC [33]. Recent studies suggest that HCC with *CTNNB1* mutations, which induces activation of the Wnt/β-catenin pathway, is characterized by reduced intratumoral T cell infiltrations [34] and can also be related to the ICI response [35]. According to the ratio of relative enhancement and visual assessment of the hepatobiliary phase, HCCs could be classified into hypo- and hyperintensity types, as well as heterogeneous and homogeneous types. In a recent small-sized study (n = 35), the heterogeneous/hyperintensity type in the baseline MRI imaging had significantly shorter PFS compared to homogeneous/hypointensity types [36].

#### 2.2.2. Perfusion Changes in Computed Tomography (CT), MRI, and Contrast-Enhanced Ultrasound (CEUS)

Changes in tumor perfusion have been observed in AB treatment in a preclinical HCC model [37], and this might be due to the immune cell infiltration by atezolizumab, as well as vascular normalization by bevacizumab. This finding implies that measuring a dynamic change in tumor perfusion might have a role in predicting the responses of AB treatment in HCC. In a recent retrospective pilot study (n = 19), perfusion change, which represents the decline in tumor-to-liver ratio in the arterial phase of CT or MRI at a mean of 9 weeks after AB treatment, was significantly associated with the disease control rate (DCR) [38]. Thus, early measures of perfusion change might help in predicting treatment response and long-term outcomes. In addition to the CT/MRI imaging, another retrospective study (n = 35) evaluated time-intensity curve (TIC) analysis using CEUS 3 to 7 days after the initial AB treatment [39]. As a result, cases without decreased blood flow showed significantly higher rates of progressive disease, compared to those with decreased blood flow. Decreased blood flow in the TIC analysis was also associated with longer PFS.

#### 2.2.3. Positron Emission Tomography-Computed Tomography (PET-CT)

Imaging characteristics on 18F-fluorodeoxyglucose PET-CT (18F-FDG-PET-CT) have demonstrated a strong association with poorly differentiated HCC, and the presence of 18F-FDG-PET/CT-positive HCC is known as an unfavorable prognostic indicator for responses to anti-HCC treatments including lenvatinib or TACE [40]. A recent retrospective study (n = 20) evaluated the tumor-to-normal liver ratio (TLR) of FDG uptake before AB treatment and found that a baseline TLR ≥ 2 was associated with early progressive disease and poor PFS, but not with OS [41].

### 2.3. Adverse Events (AEs)

The appropriate monitoring and management of AEs are important in the continuation of chemotherapy and the outcome of patients. In fact, early bevacizumab interruption within 9 weeks after treatment was related to shorter PFS and OS and was associated with AEs, including liver injury, poor oral intake, proteinuria, and ascites [42]. It was also associated with a poor mALBI grade and, importantly, it also affected the implementation of later-line treatment. The following are current data regarding AEs and their impact on the outcomes of patients in AB treatment.

#### 2.3.1. Immune-Related Adverse Events (irAEs)

Because it reinforces the immune system, ICI can cause irAEs that involve multiple organs such as the skin, gastrointestinal, respiratory, thyroid, and central nervous systems. However, it is unclear whether irAEs are associated with efficacy or survival, particularly in HCC patients receiving AB treatment. A recent retrospective study (n = 150) evaluated irAEs and their impact on the outcome of patients [43]. This study classified irAEs into endocrine, dermatologic, gastrointestinal, hepatic, hematological, pulmonary, musculoskeletal, cardiovascular, nervous system, and renal events. The authors found that total irAEs, not independent irAEs, are not associated with the ORR. However, grade 1/2 irAEs were significantly associated with favorable PFS and OS, compared to grade 3/4 irAEs or no irAEs in the multivariate analysis. Another retrospective study (n= 130) found that skin reactions were associated with longer OS [44]. These results suggest that irAEs might reflect the activation of immune function and the effectiveness of ICIs including atezolizumab, but the severe grade of irAEs might reduce this beneficial effect due to the discontinuation of treatment or their own severity.

Since most patients have liver cirrhosis (LC) or chronic hepatitis, hepatic irAEs require special attention in HCC patients receiving ICIs. Prior studies have indicated that liver damage is linked to unfavorable outcomes in cancer patients undergoing ICI therapy, and even HCC patients experiencing grade 1 or 2 liver injury during ICI treatments demonstrated a poor prognosis [45,46,47]. In patients receiving the AB treatment, liver injuries including AST, ALT, and bilirubin elevation were associated with shorter OS [44,48]. On the other hand, anti-VEGF treatment can also cause liver injury and reduce liver function as shown in a clinical trial of ramucirumab in HCC patients [49]. Therefore, further studies are needed to distinguish whether the liver injury is related to the irAE during AB treatment, and on-treatment strategies including dose modification or special management should be investigated.

#### 2.3.2. Anti-VEGF-Related AEs 

Hypertension related to anti-VEGF was linked to a better DCR and PFS in HCC patients undergoing AB therapy in a recent retrospective study (n = 286) [50]. Another study also showed that hypertension is associated with longer OS [44]. The underlying mechanism is uncertain; however, the direct relationship between VEGF inhibition and hypertension onset is evident, given the role of VEGF in maintaining normal endothelial cell function and vascular balance [51].

Proteinuria was also significantly associated with better OS in a recent Japanese real-world study (n = 286) [48]. Although its impact on bevacizumab efficacy is controversial across various types of cancer, proteinuria was correlated with VEGF signal inhibition [52].

**Table 1:** Previous studies investigating the association between clinico-radiological factors and outcome.

Clinical Markers	Related Outcomes	Study Design (n)	Reference
Viral etiology (HBV + HCV)	Favorable OS, PFS of AB compared to SOR	Phase III RCT- IMbrave150 (336)	Cheng et al. [17]
AFP reduction (≥75%) after Tx in HBV subjects	Favorable OS, PFS	Phase Ib and phase III IMbrave150 (440)	Zhu et al. [19]
Viral etiology (HBV + HCV)	Favorable ORR	Retrospective (23)	Takeda et al. [21]
Viral etiology (HBV + HCV)	Favorable OS, PFS	Retrospective (66)	Himmelsbach et al. [20]
Macrovascular invasion	Unfavorable OS	Retrospective (121)	Chon et al. [24]
Extrahepatic spread	Unfavorable PFS	Retrospective (433)	Fulgenzi et al. [25]
Child–Pugh B	Treatment discontinuation	Retrospective (28)	Tanaka et al. [30]
Child–Pugh B	Unfavorable OS, PFS	Retrospective (100)	Jost-Brinkmann et al. [26]
Child–Pugh B	Unfavorable OS, PFS	Retrospective (66)	Himmelsbach et al. [20]
Child–Pugh B	Unfavorable OS, PFS	Retrospective (457)	Tanaka et al. [27]
High ALBI and ECOG	Unfavorable OS, PFS	Retrospective (147)	de Castro et al. [28]
High ALBI	Unfavorable OS, PFS	Retrospective (50)	Sinner et al. [29]
High mALBI	Unfavorable ORR, treatment discontinuation	Retrospective (28)	Tanaka et al. [30]
High mALF score (mALBI + AFP)	Unfavorable OS, PFS	Retrospective (426)	Hatanaka et al. [32]
Worsening ALBI at wk3	Unfavorable OS	Retrospective (69)	Unome et al. [31]
Heterogeneous/hyperintensity in HBP of MRI	Unfavorable PFS	Retrospective (35)	Sasaki et al. [36]
Decline in TLR in the arterial phase of CT or MRI after Tx	Favorable DCR	Retrospective (19)	Onuoha et al. [38]
Decrease in blood flow in CEUS after Tx	Favorable DCR, PFS	Retrospective (35)	Takada et al. [39]
High tumor-to-normal ratio of FDG uptake in PET-CT	Unfavorable PFS and DCR	Retrospective (20)	Kawamura et al. [41]
Grade 1/2 irAEs	Favorable OS, PFS	Retrospective (150)	Fukushima et al. [43]
Skin reaction	Favorable OS	Retrospective (130)	Shimose et al. [44]
Liver injury	Unfavorable OS	Retrospective (130)	Shimose et al. [44]
Liver injury	Unfavorable OS	Retrospective (286)	Takaki et al. [48]
Hypertension	Favorable DCR, PFS	Retrospective (286)	Tada et al. [50]
Hypertension	Favorable OS	Retrospective (130)	Shimose et al. [44]
Proteinuria	Favorable OS	Retrospective (286)	Takaki et al. [48]

HBV, hepatitis B virus; HCV, hepatitis C virus; OS, overall survival; PFS, progression free survival; AB, atezolizumab + bevacizumab; SOR, sorafenib; RCT, randomized controlled trial; AFP, alpha-fetoprotein; Tx, treatment; ORR, objective response rate; BCLC, Barcelona Clinic Liver Cancer; ALBI, albumin–bilirubin; ECOG, Eastern Cooperative Oncology Group; mALBI, modified albumin–bilirubin; HBP, hepatobiliary phase; MRI, magnetic resonance imaging; DCR, disease control rate; CT, computed tomography; TLR, tumor–liver ratio; CEUS, contrast-enhanced ultrasonography; FDG, fluorodeoxyglucose; PET, positron emission tomography; and irAEs, immune-related adverse events.

## 3. Blood-Based Biomarkers

### 3.1. Clinically Available Blood-Based Biomarkers 

#### 3.1.1. Alpha-Fetoprotein (AFP)

Previous studies that have investigated predictive factors using blood samples are summarized in Table 2. Higher levels of AFP correlate with lower survival and higher tumor recurrence rates across different stages of HCC. Despite its association with worse outcomes, AFP has not been confirmed as a predicting factor in trials for various first-line systemic treatments. However, AFP levels above 400 ng/mL have been linked to a poorer response for ramucirumab, showing for the first time that a biomarker that can be used to select a systemic treatment in HCC [49].

Many clinical studies have investigated whether AFP can be used as a biomarker for predicting clinical outcomes in HCC patients receiving the AB treatment, with a gradual accumulation of evidence. For example, a baseline AFP level of ≥100 ng/mL was associated with poor PFS in a recent real-world study (n = 286) [50]. Furthermore, more data have reported that its dynamic change might be associated with the clinical outcome of AB treatment. A reduction in AFP levels (≥75%) at 6 weeks may serve as a potential biomarker for predicting improved OS and PFS in HCC patients receiving the AB treatment, particularly in those with HBV etiology among 440 patients enrolled in the phase Ib and III trials of IMbrave150 [19]. Another prospective study (n = 284) additionally evaluated the optimal cut-off reduction in AFP level at week six [53]. As a result, both 20% and 50% showed a significant relationship with ORR and PFS. In another retrospective study (n = 58), AFP response at 6 weeks after AB treatment was associated with the ORR, OS, and PFS [54]. Early AFP reduction at 3 weeks also predicted a better radiological response and OS in the small-sized retrospective study (n = 75) [55]. This study also showed that an AFP ratio of 1.4 or higher at 3 weeks was related to PFS. These results suggest that the dynamic change of AFP has a role as an important biomarker for early determination of treatment continuation, although this needs to be further validated.

#### 3.1.2. Protein Induced by Vitamin K Antagonist-II (PIVKA-II)

PIVKA-II is a tumor marker that is closely associated with the prognosis of HCC patients. The reduction in PIVKA-II was correlated with better ORR, OS, and PFS in HCC patients who underwent nivolumab treatment [56]. Several reports have observed an association between PIVKA-II level and prognosis in AB treatment. A recent retrospective study (n = 121) showed that a higher level of PIVKA-II at baseline (≥86 mAU/mL) was associated with poor OS and PFS [24]. A baseline PIVKA-II level of <400 mAU/mL was also associated with favorable PFS in a real-world study (n = 75) [57]. Another retrospective study (n = 69) also suggested that an early increase in PIVKA-II level was related to poor OS [31].

#### 3.1.3. C-Reactive Protein (CRP)

High levels of CRP are associated with systemic inflammation and the progression of cancer [58]. It also has an immunosuppressive effect, which might be associated with the impaired efficacy of ICIs. A previous study showed that an elevated CRP level was a significant factor for poor PFS and OS in various types of cancers treated with ICIs [59]. Scoring systems using CRP and other parameters have been developed and validated in HCC patients receiving AB. A recent study showed that CRP < 1 mg/dL and AFP < 100 ng/mL at baseline are significantly associated with OS in HCC patients receiving PD-L1 immunotherapy [60]. These two markers were subsequently used to create the CRAFITY score, and the radiological response was significantly better in a lower CRAFITY score. Another retrospective study validated the CRAFITY score as a significant factor for OS and PFS in AB-treated HCC patients (n = 89) [61]. A Japanese retrospective study (n = 297) showed that the CRAFITY score significantly predicted OS and PFS, AEs, including liver injury, loss of appetite, proteinuria, fever, and fatigue [62].

A neo-Glasgow prognostic score (GPS) was reported in HCC patients who underwent surgery associated with postoperative complications [63]. Individuals exhibiting a serum CRP concentration exceeding 1.0 mg/dl in conjunction with an ALBI grade of either two or three can be classified as having a neo-GPS score of two. A recent retrospective study validated this scoring system with 421 patients receiving AB treatment and found that it was independently related to OS and DCR [64]. Studies using CRAFITY and neo-GPS systems suggest that CRP should also be considered in the outcome prediction of AB treatment in HCC.

#### 3.1.4. Neutrophil-to-Lymphocyte Ratio (NLR) and Platelet-to-Lymphocyte Ratio (PLR)

A high NLR has been suggested to potentially serve as a marker for resistance to ICIs due to its association with intratumoral concentrations of MDSCs [65], TAMs [66], a serum cytokine profile encompassing pro-inflammatory and angiogenic cytokines [67], and the presence of tumor-infiltrating lymphocytes (TILs) [68]. Prior research has indicated that an increased NLR is linked to a worse prognosis in HCC patients undergoing nivolumab treatment [69,70]. Evidence on the role of the NLR in predicting outcomes of AB treatment in HCC patients has accumulated. An NLR > 3.21 was associated with poor ORR in a small-sized retrospective study (n = 40) [71]. Another retrospective study (n = 240) reported that an NLR ≥ 3 was not associated with the ORR, but it was associated with the cumulative discontinuation rate due to AEs, resulting in a shorter OS [72]. A German real-world study (n = 100) also showed that an NLR > 3.2 was the most significant factor predicting poor ORR and PFS [26]. Furthermore, an NLR ≥ 3 at baseline was an independent risk factor related to hyperprogressive disease (HPD) in a pilot study (n = 8) [73]. Patients with an NLR ≥ 5 had significantly poorer OS in a real-world study (n = 296) [74], and another retrospective study (n = 121) suggested that an NLR ≥ 2.5 at baseline was associated with poor OS and PFS [24]. The latter study also showed that an NLR decrease of 10% or more at the first response evaluation was an independent factor for longer OS. An NLR < 1.97 in the second course was associated with better ORR, OS, and PFS in a recent retrospective study (n = 110) [75]. An investigation to determine its optimal cut-off and mechanism should be performed in future studies. In addition to the NLR > 3, a PLR > 230 is a risk factors for poor PFS in a small-sized retrospective study (n = 48) [76]. Therefore, the NLR should be considered as a biomarker, and baseline and dynamic changes in its level should also be evaluated.

#### 3.1.5. Prognostic Nutritional Index (PNI)

The PNI can be calculated with serum albumin and the absolute count of peripheral blood lymphocytes and has been used as a prognostic marker in HCC patients who have undergone liver transplantation [77]. However, its impact on HCC patients receiving ICIs, including an AB regimen, has been unclear. A recent study showed that a high PNI of 47 or more, with an AFP level lower than 100 ng/mL, were independent factors associated with better OS and PFS in a recent retrospective study (n = 286) [78].

### 3.2. Other Blood-Based Biomarkers Based on Experimental Research

#### 3.2.1. Serum IL-6 

IL-6 is a cytokine that is elevated in hepatitis, LC, and HCC patients [79], and also has a tumor-promoting or tumorigenesis effect that might be associated with the attenuation of T cell function and recruitment [80]. In a recent study, the association between serum IL-6 and clinical outcomes in AB-treated patients was investigated prospectively (n = 165) [81]. Among the various blood-derived biomarkers, a serum level of IL-6 was significantly elevated in patients who did not achieve favorable outcomes (complete response, partial response, or stable disease for at least 6 months), and it also correlated with poor OS and PFS. This study found that high IL-6 levels correlated with decreased interferon-γ (IFN-γ) and tumor necrosis factor-α (TNF-α) secretion from CD8+ T cells, which was validated by in vitro assays showing that the treatment of IL-6 inhibited cytokine secretion and expansion of CD8+ T cells. Furthermore, patients with elevated IL-6 levels displayed a non-T-cell-inflamed immunosuppressive TME in the transcriptome analysis. Results from another prospective study (n = 64) were also compatible, which showed that higher levels of serum IL-6 correlated with a poorer ORR, OS, and PFS [82].

#### 3.2.2. Peripheral Blood PD-1 Expression on Granulocytes

In addition to the NLR, a prospective study further evaluated the expressions of PD-1 and PD-L1 of granulocytes among whole blood samples using flow cytometry in 34 patients with AB treatment [83]. Interestingly, PD-1 expression on granulocytes, but not PD-L1, was a significant factor, and a low baseline PD-1 percentage expressed on granulocytes was associated with better ORR and longer time to progression. Future translational studies investigating the characteristics of the peripheral blood immune cell population, as well as their dynamic changes, and their association with the clinical outcome in AB treatment, should be performed.

#### 3.2.3. Factors Associated with Aberrant Angiogenesis

A recent retrospective study (n = 46) evaluated the serial changes of growth factors and found that patients who initially had disease control but later progressed had significantly higher levels of VEGF-D and ANG-2 [84]. These findings suggested that increased levels of VEGF-D and ANG-2 in the serum may contribute to the resistance of AB treatment.

Insulin-like growth factor-1 (IGF-1) is also associated with angiogenesis in addition to liver function [85]. A recent study divided HCC patients receiving AB treatment into baseline IGF-1 high, normal, and low groups among 371 patients enrolled in the phase III IMbrave150 trial, and found that the low IGF-1 group showed significantly better OS and PFS [86].

Growth hormone (GH) is known to promote tumor angiogenesis [87] and is also linked to tumorigenesis or tumor-promoting effects in various types of cancers, such as breast cancer and HCC. A small-sized previous study (n = 37) evaluated the prognostic role of GH in HCC patients receiving AB, the low-GH group showed significantly better OS than the high-GH group, but not PFS [88].

**Table 2:** Previous studies investigating the association between blood-based markers and outcome.

Blood Markers	Related Outcomes	Predictive/Prognostic	Study Design (n)	Reference
AFP ≥ 100 ng/mL	Unfavorable PFS	Prognostic	Retrospective (286)	Tada et al. [50]
AFP < 100 ng/mL	Favorable OS, PFS	Prognostic	Retrospective (485)	Tada et al. [78]
AFP reduction (≥75%, 6 wks) in HBV subjects	Favorable OS, PFS	Prognostic	Phase Ib and phase III IMbrave150 (440)	Zhu et al. [19]
AFP reduction (≥50% or 20%, 6 wks)	Favorable ORR, PFS	Predictive, prognostic	Prospective (284)	Tamaki et al. [53]
AFP reduction, 6 wks	Favorable ORR, OS, PFS	Predictive, prognostic	Retrospective (58)	Kuzuya et al. [54]
Early AFP reduction, 3 wks	Favorable ORR, OS, PFS	Predictive, prognostic	Retrospective (75)	Campani et al. [55]
PIVKA-II ≥ 186 mAU/mL	Unfavorable OS, PFS	Prognostic	Retrospective (121)	Chon et al. [24]
PIVKA-II ≥ 400 mAU/mL	Unfavorable PFS	Prognostic	Retrospective (75)	Ochi et al. [57]
Early increase in PIVKA-II	Unfavorable OS, PFS	Prognostic	Retrospective (69)	Unome et al. [31]
High CRAFITY (CRP + AFP) score	Unfavorable OS, PFS, frequent AEs	Prognostic	Retrospective (297)	Hatanaka et al. [62]
High CRAFITY (CRP + AFP) score	Unfavorable OS, PFS	Prognostic	Retrospective (89)	Teng et al. [61]
High Neo-GPS (CRP + ALBI) score	Unfavorable OS, DCR	Predictive, prognostic	Retrospective (421)	Tada et al. [64]
NLR > 3.21	Unfavorable ORR	Predictive	Retrospective (40)	Eso et al. [71]
NLR ≥ 3	Unfavorable OS, treatment discontinuation	Prognostic	Retrospective (240)	Tada et al. [72]
NLR > 3.2	Unfavorable ORR, PFS	Predictive	Retrospective (100)	Jost-Brinkmann et al. [26]
NLR ≥ 3	Hyperprogressive disease	Prognostic	Retrospective (8)	Maesaka et al. [73]
NLR ≥ 5	Unfavorable OS	Prognostic	Retrospective (296)	Wu et al. [74]
NLR ≥ 2.5	Unfavorable OS, PFS	Prognostic	Retrospective (121)	Chon et al. [24]
NLR decrease ≥ 10% (at 1st response evaluation)	Favorable OS	Prognostic	Retrospective (121)	Chon et al. [24]
NLR ratio at second course < 1.97	Favorable ORR, OS, PFS	Predictive, prognostic	Retrospective (110)	Matoya et al. [75]
NLR > 3, PLR > 230	Unfavorable PFS	Prognostic	Retrospective (48)	Wang et al. [76]
PNI (albumin + peripheral lymphocyte counts) ≥ 47	Favorable OS, PFS	Prognostic	Retrospective (286)	Takaki et al. [78]
High serum interleukin-6	Unfavorable DCR, OS, PFS	Predictive, prognostic	Prospective (165)	Yang et al. [81]
High serum interleukin-6	Unfavorable ORR, OS, PFS	Predictive, prognostic	Prospective (64)	Myojin et al. [82]
Low baseline PD-1% on granulocytes	Favorable ORR, TTP	Predictive	Prospective (34)	Giovannini et al. [83]
Elevated VEGF-D and ANG-2	Durable disease control	Predictive	Retrospective (46)	Yang et al. [84]
Low IGF-1	Favorable OS, PFS	Prognostic	Phase III IMbrave150 (371)	Kaseb et al. [86]
Low growth hormone	Favorable OS	Prognostic	Prospective (37)	Mohamed et al. [88]
High anti-drug antibodies, 3 wks	Unfavorable OS, PFS	Prognostic	Prospective (174)	Kim et al. [89]
High ctDNA	Unfavorable ORR, OS, PFS	Predictive, prognostic	Retrospective (85)	Matsumae et al. [90]
TERT mutation within ctDNA	Unfavorable OS	Prognostic	Retrospective (85)	Matsumae et al. [90]
Low CXCL9 within ctDNA	Unfavorable DCR	Predictive	Retrospective (29)	Hosoda et al. [91]

AFP, alpha-fetoprotein; wks, weeks; PFS, progression-free survival; OS, overall survival; ORR, objective response rate; AEs, adverse events; DCR, disease control rate; HBV, hepatitis B virus; PIVKA-II, protein induced by vitamin K antagonist-II; GPS, Glasgow prognostic score; ALBI, albumin–bilirubin; NLR, neutrophil–lymphocyte ratio; PLR, platelet–lymphocyte ratio; PNI, prognostic nutritional index; PD-1, programmed cell death-1; VEGF, vascular endothelial growth factor; ANG-2, angiopoietin-2; IGF-1, insulin-like growth factor-1; ctDNA, circulating tumor DNA; TERT, telomerase reverse transcriptase; and CXCL9, chemokine ligand9.

## 4. Tissue-Driven Biomarkers

### 4.1. Biomarkers Related to Immune Cells 

#### 4.1.1. PD-L1 Expression

Previous studies that have investigated predictive factors using tissue samples are summarized in Table 3. In the tumor and adjacent liver tissues of HCC, PD-L1 can be expressed by tumor cells, Kupffer cells, hepatocytes, and sinusoidal cells [92]. Although its expression is variable across the studies and antibody clones used for immunohistochemistry (IHC), its expression within tumor tissues has been considered to be associated with macrovascular invasion, poor differentiation, high AFP levels, and poor prognosis in HCC, as reported previously [93,94]. Its impact on the efficacy of ICI treatment in HCC patients is controversial. For example, PD-L1 expression measured by IHC on tumor cells did not affect the outcome of HCC patients treated with nivolumab [95], but the baseline combined positive score (CPS) of PD-L1 expression was related to ORR in pembrolizumab-treated patients [96]. In the phase III IMbrave150 trial, PD-L1 expression (clone SP263) with a CPS ≥ 1% was associated with the benefits of the AB treatment in terms of PFS and ORR, compared to the sorafenib treatment [17]. However, another experimental study included 358 patients enrolled in the phase Ib and III IMbrave150 trial, which also used an SP263 clone for IHC, did not observe a difference in ORR [97]. Therefore, further studies are needed to validate the role of PD-L1 expression measured by IHC as a biomarker for AB treatment in HCC. Of note, the standardization for the antibody clone and staining methods, and adjustment of the cut-off value are mandatory as it is the key target molecule of AB treatment.

#### 4.1.2. Immune Cell Infiltrations within Tumor Tissues

Although immune cells including various subsets of T cells, TAMs, and MDSCs among the TME of HCC might be closely related to the responses to ICI treatment, limited data have been reported, probably due to the difficulty in obtaining tumor tissues. In nivolumab-treated HCC patients, higher baseline CD3+ and CD8+ TILs measured by IHC were related to better OS [69]. Furthermore, higher CD3+ and CD8+ TILs 6 weeks after tremelimumab treatment for HCC were correlated to the ORR [98], which implicates the role of tumor biopsy and histologic examinations for immune cell populations before and after ICI treatment.

Using multiplex IHC staining, a previous study using samples from 358 patients enrolled in the phase Ib and phase III IMbrave150 trial showed that higher infiltrations of CD3+ T cells, CD8+ T cells, MHC-1+ tumor cells, and CD3+granzyme B+ T cells, within tumor tissues were associated with the ORR of AB treatment [97]. They also used xCell deconvolution analysis from transcriptome data and observed that a higher presence of CD8+ T cells, CD4+ T cells, Tregs, B cells, and dendritic cells (DCs) is associated with better ORR and PFS. Further examinations evaluating various immune cell populations within tumor tissues before and after AB treatment should be performed. In addition, the baseline evaluation and monitoring of the peripheral immune cell population should also be considered in future studies.

### 4.2. Atezolizumab-Bevacizumab Response Signature

The development of novel signatures by tumor tissues that can predict responses should also be investigated. Using bulk RNA sequencing analyses of tumor tissues, the authors built an atezolizumab–bevacizumab response signature (ABRS) that contains genes related to pre-existing immunity, including genes associated with high PD-L1 and effector T cell signatures using samples from patients enrolled in the phase Ib and phase III IMbrave150 trial. As a result, a high ABRS was significantly associated with better ORR and PFS [97].

## 5. Novel Biomarkers

### 5.1. Anti-Drug Antibodies (ADA)

Atezolizumab treatment has been reported to induce ADA [99]. Consequently, this might reduce the level of atezolizumab, but the impact on clinical efficacy has been unclear. Serum ADA can be measured by enzyme-linked immunosorbent assays. A recent prospective multicenter cohort study (n = 174) showed that a higher ADA level in AB-treated HCC patients at 3 weeks was associated with poor PFS and OS [89]. A high level of ADA against atezolizumab at 3 weeks after initial treatment was found in 17.4% of the patients, whereas 82.6% of the patients had low or negative results. The levels of ADAs were negatively correlated with the level of atezolizumab. A high-ADA group had significantly lower OS and PFS in discovery (HR = 2.84 and 3.30, respectively) and validation (HR = 2.52 and 5.81, respectively) studies. Furthermore, patients with higher ADA levels showed lower proliferation and cytokine-secreting functions of T cells, suggesting that ADA attenuated the antitumor immune response. Future studies might be needed to validate this study.

### 5.2. Liquid Biopsy 

#### 5.2.1. Circulating Tumor DNA (ctDNA) Levels and *TERT* Mutation

The number of ctDNA can be correlated with tumor stage and prognosis in HCC, and a recent study (n = 85) evaluated its association with prognosis in patients receiving AB [90]. As a result, a higher level of ctDNA was correlated with poorer ORR, PFS, and OS. When ultradeep sequencing was performed, the *TERT* promoter, tumor protein 53 (*TP53*), and catenin beta 1 (*CTNNB1*) were frequently mutated, but *TERT* ctDNA mutation was an independent factor predicting poor OS. *CTNNB1* mutation was not a significant factor, as found in another study [100]. These findings imply that measuring and profiling ctDNA should be researched with a larger sample size in future studies.

#### 5.2.2. *CXCL9* within ctDNA

Litchfield and colleagues examined whole-exome and transcriptome data from over 1000 patients undergoing treatment with ICIs and found that a high expression of *CXCL9* is among the most significant factors predicting a positive response to ICI therapy [101]. This might be due to the role of *CXCL9* in attracting cytotoxic CD8+ T cells to the tumor site [102]. In a recent Japanese retrospective study (n = 29), authors performed a cytokine array analysis using ctDNA and found that low *CXCL9* levels within the ctDNA were associated with early PD in discovery and validation cohorts receiving AB [91].

## 6. Potential but Unproven Markers

### 6.1. Sarcopenia and Obesity

Sarcopenia refers to the gradual decline in both the amount and power of skeletal muscles and is associated with the prognosis in HCC patients who underwent surgical resection [103] and systemic therapies including sorafenib or lenvatinib [104,105]. In a small-sized retrospective study, there was no association between sarcopenia and prognosis in AB-treated HCC patients [106]. The association between body mass index (BMI) and the clinical outcome of ICI treatment is controversial across the cancer types and studies, and the relationship between BMI and immunotherapy in HCC has not been researched extensively. One study has shown that a BMI of 25 or higher is linked to better OS, but not PFS, in patients treated with PD-1 antibody-based therapies [107]. A recent study investigated whether high BMI is associated with the clinical outcome in patients with the AB treatment, but there was no significance in OS, PFS, and ORR [108]. Nevertheless, more data are needed to confirm the role of sarcopenia and obesity in the prognosis of HCC patients treated with AB.

### 6.2. Tumor Mutational Burden (TMB)

TMB has been studied across various human cancers, and a high TMB has been considered to reflect a high load of tumor neoantigens and responses to the ICI treatment for cancers, which might be due to more recognition of tumor cells by T cells and enhanced antitumor immune responses [109]. However, its impact on HCC patients receiving ICIs has been controversial. A previous study showed that the frequency of patients with a high TMB was very low (0.8%, 6 of 755 patients with HCC), and was not related to the response rate [110]. In patients receiving camrelizumab and afatinib, TMB was associated with the ORR, although the sample size was small [111]. In a recent experimental study of AB treatment, TMB was not associated with PFS and ORR [17]. However, considering its impact on antitumor immune responses, larger and multicenter studies are needed to elucidate the role of TMB in HCC patients receiving the AB treatment. Importantly, the methods of sequencing and the cut-off value of TMBs should be standardized in future studies.

### 6.3. Gene Mutations

Alterations in genes related to the immune function or oncogenic pathways in tumor tissues have been studied in many types of cancers including HCC. However, there is a lack of data on HCC patients receiving AB treatment. The immune-exclusion class characterized by *Wnt/CTNNB1* mutation has been known to be associated with resistance to ICI treatment in HCC [112]. In a previous study, a tumor biopsy occurred before AB treatment, and expression levels of glutamine synthetase and β-catenin, which are markers of Wnt/β-catenin signal activation, were measured, but there was no difference in PFS and ORR between activation and inactivation groups [113]. *TP53* gene mutations, which are related to an immunosuppressive TME in HCC [114], have not been studied in patients receiving ICI or AB treatments for HCC.

Previous studies showed that telomerase reverse transcriptase (*TERT)* promoter mutations are associated with poor outcomes in various types of cancers, and are particularly related to the epithelial–mesenchymal transition [115] and PD-L1 expression [116]. This might be correlated with a higher TMB and better response to ICIs [115]. Furthermore, antigens from the TERT protein have been considered to be immunogenic and recognized by T cells, which is the main effector of ICI treatment [117]. Of note, a recent experimental study showed that a *TERT* promoter mutation is associated with the clinical benefit of AB treatment compared to sorafenib in terms of OS and PFS [97]. Further mechanistic and validation studies in AB-treated HCC patients should be performed.

### 6.4. Gut Microbiome

It has been reported that the diversity, composition, and function of the gut microbiome might be associated with immune responses. Zheng et al. investigated whether the gut microbiome has an impact on the responsiveness of anti-PD-1 treatment in patients with HCC [118]. As a result, responders had a higher richness of taxa and gene counts compared to non-responders, and diversity and stability were maintained after anti-PD-1 treatment whereas it is not maintained in non-responders. However, their impact on anti-VEGF treatment, as well as their role as a biomarker in the AB treatment of HCC patients, still needs to be clarified.

## 7. Discussion

The IMbrave150 trial represents a significant milestone in the systemic treatment for advanced HCC and has contributed to further investigations and approvals of ICI-based combination treatments. As confirmed in the meta-analysis of various types of cancers, including HCC, ICI treatment can increase the chance of a complete response compared to conventional treatments [119]. Moreover, the addition of locoregional treatments, including transarterial radioembolization to the ICI-regimen has also been tried to maximize the clinical benefit [120]. Nevertheless, a considerable number of patients do not respond to the treatment, about 70% [121], indicating the need to identify predictive biomarkers to guide initial and on-treatment decisions in HCC patients receiving AB.

Considering the mechanism of action for AB, it is crucial to develop biomarkers that accurately reflect the interplay between angiogenesis and immunosuppression within the TME. Of note, VEGF not only fosters angiogenesis in tumors but also establishes an immunosuppressive TME by attracting and inducing immunosuppressive cells, such as Tregs, TAMs, and MDSCs [11]. Moreover, VEGF hampers DC differentiation and maturation, as well as effector T cell proliferation, ultimately weakening T cell priming and targeting cell elimination [11]. In addition, VEGF drives the T cell exhaustion-specific program, which upregulates transcription factor TOX in the TME [122]. Thus, blocking VEGF signaling not only inhibits intra-tumoral angiogenesis and normalizes tumor vasculature but also transforms the TME from immunosuppressive to immune-active, which might enhance the efficacy of ICIs. These potential synergisms might improve AB efficacy in HCC compared to monotherapy-based ICIs such as nivolumab and pembrolizumab.

The analysis of biomarkers in HCC has been challenging in various treatment modalities, and no standard or definite predictive biomarkers have been identified for patients receiving AB treatment. The unique environment involving hepatitis and/or cirrhosis with different etiologies in HCC might contribute to this challenge. To predict treatment response more accurately, an integrative approach that combines clinical, histopathology, imaging, and circulating markers is necessary. The fact that this regimen contains not only ICI but also VEGF inhibitors, should be considered when developing biomarkers to optimize therapeutic strategies. Furthermore, baseline biomarkers can help identify patients who are likely to respond to treatment, whereas dynamic biomarkers, which are assessed during the therapy, can provide crucial information about treatment efficacy and the potential need for adjustments.

There are some limitations to the current review. Because many of the previous reports we have reviewed were small-sized, retrospective studies, there is a possibility of bias, misrepresentation, or inconsistency. Therefore, data should be interpreted cautiously, and most biomarkers need to be validated in larger, prospective future studies. In addition, systemic treatment and immunotherapy for HCC is a rapidly evolving field; reviews regarding its prognostic or predictive biomarkers can also quickly become outdated, necessitating regular updates.

## 8. Concluding Remarks

The increasing availability of real-world data from patients treated with AB is expected to facilitate the development of more accurate predictive biomarkers, ultimately improving the clinical outcome of first-line systemic treatment for HCC. In addition to the examination of peripheral blood, tumor tissues, and clinical information, liquid biopsy or microbiome analysis could also play a role in predicting responses to AB and should be investigated in future studies. Importantly, the development of biomarkers for the AB treatment of HCC should consider integrative and dynamic approaches. This comprehensive strategy may lead to better patient selection, more precise prediction of treatment response, and optimized therapeutic strategies, ultimately resulting in improved patient outcomes. Furthermore, validation tools other than patient cohorts, such as organoids or patient-derived xenograft models, could be used in the development of biomarkers in AB treatment and could also be applied to the agents for novel therapeutic targets of HCC.

## Figures and Tables

**Table 3:** Previous studies investigating the association between tissue-based markers and outcome.

Tissue Markers	Related Outcomes	Study Design (n)	Reference
PD-L1 expression CPS ≥ 1% (IHC, clone SP263)	Favorable ORR, PFS of AB compared to SOR	Phase III RCT- IMbrave150 (336)	Cheng et al. [17]
Signature associated with the PD-L1 expression (RNA sequencing)	Favorable ORR, PFS	Phase Ib and phase III IMbrave150 (358)	Zhu et al. [97]
*TERT* promoter mutation (whole exome sequencing)	Favorable ORR, PFS of AB compared to SOR	Phase Ib and phase III IMbrave150 (358)	Zhu et al. [97]
High atezolizumab–bevacizumab response signature (ABRS) -PD-L1 and effector T cell signatures (RNA sequencing)	Favorable ORR, PFS	Phase Ib and phase III IMbrave150 (358)	Zhu et al. [97]
High infiltration of CD3/8+ T cells, Granzyme+ T cells, and MHC-I+ tumor cells (IHC)	Favorable ORR	Phase Ib and phase III IMbrave150 (358)	Zhu et al. [97]
High expression of CD4/8+ T cells, Treg cells, B cells, and DCs (RNA sequencing)	Favorable ORR, PFS	Phase Ib and phase III IMbrave150 (358)	Zhu et al. [97]

PD-L1, programmed cell death-ligand 1; CPS, combined positive score; IHC, immunohistochemistry; ORR, objective response rate; PFS, progression-free survival; AB, atezolizumab + bevacizumab; SOR, sorafenib; TERT, telomerase reverse transcriptase; MHC, major histocompatibility complex; Treg, regulatory T; and DC, dendritic cell.

## Data Availability

The data that support the findings of this review are available from the corresponding author upon reasonable request.
